# Popular infection prevention myths related to single-use plastics

**DOI:** 10.1017/ash.2026.10786

**Published:** 2026-07-24

**Authors:** Pamela S. Lee, Shira Abeles, Preeti Mehrotra, Trini Mathew, Preeti Jaggi, Shreya Doshi, Vincent Hsu, Hardeep Singh

**Affiliations:** 1 Division of Infectious Diseases, Harbor-UCLA Medical Center and the Lundquist Institute at Harbor-UCLA, Torrance, CA, USA; 2 Department of Medicine, David Geffen School of Medicine at UCLA, Los Angeles, CA, USA; 3 Division of Infectious Diseases and Global Public Health, University of California San Diego, San Diego, CA, USA; 4 Division of Infectious Diseases, Beth Israel Deaconess Medical Center, Harvard Medical School, Boston, MA, USA; 5 HealthTAMCycle3 PLLC, Troy, MI, USA; 6 Department of Pediatrics, Division of Infectious Diseases and Children’s Healthcare of Atlanta, Emory University, Atlanta, GA, USA; 7 Division of Infectious Diseases, Children’s National Hospital, Washington, DC, USA; 8 Department of Pediatrics, George Washington University, Washington, DC, USA; 9 AdventHealth, Altamonte Springs, FL, USA; 10 Loma Linda University School of Medicine, Loma Linda, CA, USA; 11 Safety, Quality & Well-Being Institute at Houston Methodist, Houston, TX, USA

## Introduction

Health impacts from plastic chemicals are estimated to result in hundreds of billions of dollars in healthcare costs annually.^1^ Emerging data also suggest adverse health impacts of microplastics and nanoplastics on human tissues.^2^ Plastic production, use, and disposal lead to significant greenhouse gas emissions, and are projected to account for 4.5% of all global greenhouse gas emissions by 2,060.^3^ Despite these human and planetary health impacts, the production and use of single-use plastics continue to grow exponentially globally.^3^

U.S. hospitals produce over 5.9 million tons of waste each year, including 1.7 million tons of plastic waste.^4^ Large-scale information on healthcare waste composition is limited. However, healthcare waste audits show that single-use disposable items used for infection prevention and control (IPC) contribute significantly to plastic waste.^5^ IPC practices can create significant healthcare waste as a byproduct in the quest to ensure patient safety and achieve zero-harm.^6^ Moreover, following ambiguous or non-evidence-based practices could lead to unwarranted, excessive use of single-use plastics and unintentionally perpetuate their use.^7^ Many such practices are misconceived and persist as “myths,” ie, beliefs held to be true despite substantial evidence to the contrary.^8^ In an effort to bust myths related to IPC and single-use plastics, we assembled a group of IPC and patient safety experts to develop a list of common myths and examined them in relation to current evidence. We subsequently surveyed health professionals with expertise in infectious diseases/IPC regarding their perceptions of common practices that may be considered myths regarding the use of single-use plastics in IPC.

## Methods

Using an informal network of expert presenters at national sustainability conferences, we convened a group of 8 experts across several disciplines: infectious disease, infection prevention, epidemiology, health care quality, patient safety, and healthcare sustainability. The group iteratively identified a list of 8 myths based on the current literature, their observations of healthcare practices across various clinical settings, and their experience in multiple healthcare settings (acute care hospitals, rehabilitation hospitals, skilled nursing facilities, clinics) and hospital types (academic, community, safety-net). We then developed a brief, one-question survey that asked healthcare professionals to select from a predefined list of common myths related to single-use plastics in IPC. We asked respondents to select up to 5 practices from the predetermined list of 8 myths based on how common and impactful they perceived that myth to be based on their own experience. We included an “other” category to allow respondents to enter their own myth if desired. Respondents were not asked to rank the myths. We distributed the survey via several listservs that included health professionals from across the US, mostly consisting of physicians and nurses with an interest in infectious diseases, IPC, and sustainability. Proposed myths included the use of: 1) disposable vs reusable isolation gowns, 2) disposable vs reusable stethoscopes for contact isolation rooms, 3) gloves vs hand hygiene for universal precautions, 4) double vs single peel pouches for sterile equipment, 5) plastic draping for clean equipment, 6) single use vs reusable non-critical devices, 7) plastic vs alternatives for providing beverages and dispensing medications, and lastly 8) whether patients had preferences for single-use disposable plastics vs reusables.

## Results

**Figure 1.**
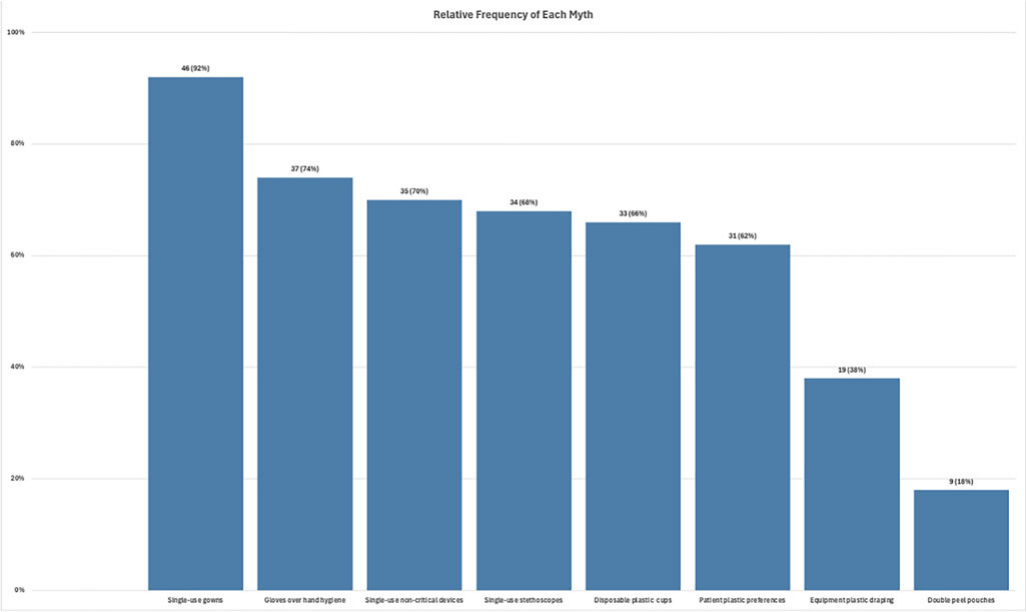
Results for our survey titled “Identifying Popular Myths in Infection Prevention and Single-Use Plastics”. Actual text included in the survey with relevant abbreviation in brackets: *Use of double peel pouches is preferred over single peel pouches when reprocessing certain sterile equipment (forceps and tongs)* [double peel pouches]; *clean equipment (wheelchairs, IV poles) must be draped in plastic to indicate that it is clean.* [Equipment plastic draping]; *patients prefer single-use disposable plastic items to reusable alternatives.* [Patient plastic preferences]; *disposable plastic cups are required for patient beverages and medication dispensing, rather than cups made of paper or reusable materials.* [Disposable plastic cups]; *single-use disposable stethoscopes must be used for patients in contact isolation rather than reusable stethoscopes that are cleaned with room turnover and left in the room.* [Single-use stethoscopes]; *non-critical devices (BP cuffs, pulse oximeters) are safer when they are single use compared to FDA-approved reprocessed items obtained via 3rd party reprocessors.* [Single-use non-critical devices]; *use of gloves is required to examine patients who need only universal precautions, rather than use of hand hygiene alone.* [Gloves over hand hygiene]; *single-use gowns are preferable to reusable gowns for isolation precautions.* [Single-use gowns].

The survey was distributed to a convenience sample of 317 health care professionals, of which 51 responded (response rate 16%). All respondents selected 5 myths in their response—ie, no respondent chose less than 5 answers in their response. Among the 8 myths (Figure 1), the most frequently selected myth was “Single-use gowns are preferable to reusable gowns for isolation precautions”—selected by 47/51 respondents (92%). The next most frequent myth was “Use of gloves is required to examine patients who need only universal precautions, rather than use of hand hygiene alone” (38/51; 75%). The least frequently selected myth was “Use of double peel pouches is preferred over single peel pouches when reprocessing certain sterile equipment (forceps, tongs) (10/51; 11%).

New myths suggested by respondents related to topics, such as contact precautions for COVID, need for single-use laryngoscope handles and blades, and single-use anesthesia machine circuits; use of plastic resealable bags to maintain sterility. Each of these was suggested by a single person and discussed by the team, after which they were excluded from consideration for the top 5.

## Discussion

Despite increasing evidence of environmental and health risks associated with single-use plastics, their use remains prevalent in healthcare. Practices favoring single-use plastics may arise from healthcare myths—ie, beliefs perpetuated despite lack of evidence or contrary evidence. We surveyed healthcare professionals with expertise in infectious diseases/IPC about which single-use plastic myths are most common in healthcare. The most common identified myths involved the use of single-use disposable gowns and gloves.

The myths identified in our study could be responsible for a significant excess in GHG emissions and solid waste. While our sample size was limited, and we did not assess the various practices and policies from which our respondents derived their experiences, these data provide a foundation for additional work to evaluate and mitigate the plastic waste potentially associated with these myths in healthcare. As a next step, policymakers and accrediting organizations could use this information to produce “myth-busting” guidance for hospitals, while keeping in mind facility-specific factors that may impact implementation of this guidance. Based on our results, such guidance could initially focus on appropriate glove use and IPC considerations associated with reusable isolation gowns. The findings also identify the need for professional societies to develop and implement clearer IPC guidelines to reduce pollution from single-use plastics in healthcare.^9^
